# Dysphagia in cervical dystonia patients receiving optimised botulinum toxin therapy: a single-center retrospective cohort study

**DOI:** 10.1007/s00702-020-02220-z

**Published:** 2020-06-25

**Authors:** Anna Kutschenko, Martin Klietz, Lejla Paracka, Katja Kollewe, Annika Schulte-Sutum, Theda Janssen, Christoph Schrader, Florian Wegner, Dirk Dressler

**Affiliations:** Department of Neurology, Hannover Medical School, Carl-Neuberg-Str. 1, 30625 Hannover, Germany

**Keywords:** Botulinum toxin therapy, Cervical dystonia, Dysphagia, Adverse effects, Risk factors, Frequency

## Abstract

To explore the correlations of botulinum toxin (BT) therapy with dysphagia, we wanted to study a group of cervical dystonia (CD) patients with optimised BT therapy during a prolonged period of time to record their dysphagia frequency, severity and duration, to study potential risk factors and try to avoid it by BT application with ultrasound guidance. BT therapy of 75 CD patients (23 males, 52 females, age 60 ± 12 years, BT total dose 303.5 ± 101.5 uMU) was retrospectively analysed for 1 year. BT therapy was optimised prior to the observation period. Dysphagia was noticed by one fifth of the patients. In those patients, it only occurred in about one third of the injection series. It was never associated with a functional deficit and lasted several days to 2 weeks. It was not related to patient age or gender, BT total dose, BT dose in the sternocleidomastoid muscle, BT dose in the sternocleidomastoid and scalenii muscles, by BT therapy with bilateral sternocleidomastoid muscle injections or BT therapy with abobotulinumtoxinA. Ultrasound guidance was not able to prevent it. Further prospective studies will be necessary to study underlying dystonia associated swallowing abnormalities as a potentially predisposing factor.

## Introduction

Cervical dystonia (CD) is the most common focal primary dystonia and is associated with decreased quality of life (Drexel et al. 2020). First line treatment of CD are intramuscular injections of botulinum toxin (BT). Their most common adverse effect is dysphagia. However, many aspects of this dysphagia still remain unclear including its frequency, severity, duration, reproducibility, its potential risk factors, and strategies to avoid it (Blackie and Lees 1990; Anderson et al. 1992; Comella et al. 1992, 2011; Poewe et al. 1998; Truong et al. 2010). We wanted to retrospectively study a group of CD patients with optimised BT therapy during a prolonged period of time, record the frequency, severity, duration, and reproducibility of their dysphagia, study potential risk factors, and evaluate BT application with ultrasound guidance.

## Methods

### Design

This was a retrospective chart review study monitoring the occurrence of dysphagia during a period of 12 months under BT therapy. It took place at the Movement Disorders Section, Department of Neurology, Hannover Medical School from July 2018 to July 2019.

### Ethics

The study was approved by the local Ethics Committee of Hannover Medical School (No. 7927_BO_K_2018). Patients gave written informed consent to the analysis and publication of their digital BT therapy data.

### Patients

This study included 75 patients with CD (23 males, 52 females, age 60 ± 12 years) receiving regularly BT therapy of which at least one was performed under ultrasound guidance during the observation period. To reduce selection bias, only patients were included whose BT doses and other treatment parameters were previously optimised and had not been changed for at least 2 injection series prior to the observation period. Besides, it was checked that none of the patients had a history of dysphagia before BT therapy was started and that none of the patients had another diagnosis that could potentially induce dysphagia. At each injection series, patients were asked about their swallowing function. Dysphagia was defined as any reported change of swallowing in relation to the respective injection cycle.

### Study parameters

Study parameters recorded included age, gender, dates of BT application, occurrence of dysphagia, injection scheme including all target muscles, target muscle doses, total BT dose, and guidance technique.

### BT drugs

Patients were treated with onabotulinumtoxinA (ONA, Botox^®^, Allergan Pharmaceuticals Ireland, Westport, Ireland), incobotulinumtoxinA (INCO, Xeomin^®^, Merz Pharmaceuticals GmbH, Frankfurt/Main, Germany) or abobotulinumtoxinA (ABO, Dysport^®^, Ipsen Pharma GmbH, München, Germany). INCO 100MU and ONA 100MU were reconstituted with 2.5 ml normal saline, ABO 500MU with 5.0 ml normal saline. Since these manufactured BT products are not formulated in the same way and their units are not determined with the same protocols, it is difficult to interconvert them other than in an arbitrary manner. Hence, for comparison of different BT drugs, doses are given as unified mouse units (uMU).Owing to the fact that most patients were treated with ONA or INCO, these doses were left unmodified (conversion ratio 1:1). Doses of ABO were divided by 2.5, a well-established conversion ratio between ABO and ONA as well as INCO (Wohlfarth et al. 2008; Frevert 2015; Albrecht et al. 2019). 1uMU was equal to 1MU of ONA, 1MU of INCO and 2.5MU of ABO. All BT injections were performed using a 27G needle of either 25 mm or 40 mm length with a 1.0 ml syringe.

### Injectors

All injectors performing the BT therapy are experienced in BT therapy and specialised in movement disorders. They were trained to perform ultrasound guided intramuscular injections and used them for at least 6 months prior to the study.

### Ultrasound guidance

Ultrasound guidance was performed with a portable ultrasonic device (MyLab25Gold, Esaote Deutschland GmbH, Köln, Germany) with a 18 MHz probe (LA435, Esaote Deutschland GmbH, Köln, Germany) covered by a sterile transducer cover with ultrasonic transmission gel inside. The probe was placed on the skin so that the target muscle was visible in the centre of the ultrasound screen.

### Anatomical guidance

Anatomical guidance was based on palpation and anatomical landmarks.

### Statistics

Data are shown as mean ± standard deviation. To check for significance of normally distributed data with equal variances the unpaired student’s *t* test was used. Otherwise, the Wilcoxon-ranksum-test was used. To test the association between two different groups, the chi-square test was used. The significance level was defined as 5% (*p* < 0.05). The statistics were performed with Microsoft Excel 2017 and MATLAB R2018a.

## Results

### Frequency

Fifteen patients (20%) of the 75 patients studied experienced dysphagia during the observation period.

### Reproducibility

In the group of patients with dysphagia (63 BT injections), it occurred after 32 ± 19% of BT applications (20 BT injections). In 12 patients dysphagia occurred only once during the observation period, in 1 patient twice and in two patients three times.

### Severity

If dysphagia occurred, it was reported only as a change of the sensation of swallowing. In none of these events eating habits had to be changed or physicians had to be consulted. There was no aspiration, asphyxia or pneumonia. None of the patients stopped BT therapy because of dysphagia.

### Duration

The duration of symptoms was between a few days to 2 weeks, before it spontaneously disappeared.

### Predictability

Potential risk factors related to dysphagia are shown in Table 1. BT total doses in patients with dysphagia (63 BT injections) were 269.9 ± 69.9 uMU, in patients without dysphagia (263 BT injections) 315.5 ± 103.4 uMU (Wilcoxon-ranksum-test, *p* < 0.001). BT doses in the sternocleidomastoid muscle were 37.9 ± 16.5 uMU in patients with dysphagia and 40.8 ± 19.2uMU in patients without dysphagia (Wilcoxon-ranksum-test, *p* = 0.242). BT doses in the sternocleidomastoid (SCM) and scalenii muscles were 35.2 ± 15.5 uMU in patients with dysphagia and 37.9 ± 18.5 uMU in patients without dysphagia (Wilcoxon-ranksum-test, *p* = 0.226). Other factors including patient age and gender and BT therapy with bilateral SCM injections were not significantly associated with the occurrence of dysphagia.

**Table 1:** Potential risk factors for dysphagia in botulinum toxin therapy

Factor	Patients with dysphagia	Patients without dysphagia	Significance
Female sex [*n*]	13	39	Chi-square test *p* = 0.104
Male sex [*n*]	2	21	
Patient age [years]	56.4 ± 11.5	61 ± 12	Unpaired *T*-test *p* = 0.191
Total number of injection series [*n*]	63	263	N/A
Injection series per patient and year [*n*]	4.1 ± 0.8	4.1 ± 0.8	Unpaired *T*-test *p* = 0.979
BT total dose [uMU]	269.9 ± 69.9	315.5 ± 103.4	Wilcoxon-ranksum-test *p* < 0.001
BT dose in SCM [uMU]	37.9 ± 16.5	40.8 ± 19.2	Wilcoxon-ranksum-test *p* = 0.242
BT dose in SCM + SCA [uMU]	35.2 ± 15.5	37.9 ± 18.5	Wilcoxon-ranksum-test *p* = 0.226
BT therapy with unilateral SCM injections [*n*]	41	159	Chi-square test *p* = 0.498
BT therapy with bilateral SCM injections [*n*]	22	104	
BT therapy with ultrasound guidance [*n*]	21	96	Chi-square test *p* = 0.638
BT therapy with anatomical guidance [*n*]	42	167	
BT therapy with ONA + INCO [*n*]	55	228	Chi-square test *p* = 0.898
BT therapy with ABO [*n*]	8	35	

ABO abobotulinumtoxinA, INCO incobotulinumtoxinA, ONA onabotulinumtoxinA, BT botulinum toxin, SCA scalenii muscles, SCM sternocleidomastoid muscle

### Ultrasound guidance

In patients without dysphagia, ultrasound guidance was used in 96 injections and anatomical guidance in 167 injections. In patients with dysphagia 21 injections were done with ultrasound guidance and 42 injections with anatomical guidance (Chi-square test, *p* = 0.638). When ultrasound guidance was used in patients with dysphagia, 3 BT applications induced dysphagia and 18 applications did not evoke it. When anatomical guidance was used in patients with dysphagia, this side effect resulted after 17 applications and dysphagia did not occur in 25 applications (Chi-square test, *p* = 0.035).

## Discussion

### Frequency

One fifth of our patients reported dysphagia. For none of them dysphagia was more than a mere observation without any functional impairment. In the literature, frequency data on dysphagia are contradictory and range from 6 to 44% of injection series (Blackie and Lees 1990; Anderson et al. 1992; Comella et al. 1992, 2011; Poewe et al. 1998; Truong et al. 2010). This may be caused by pre-described treatment protocols not allowing adequate individualisation and optimisation of the treatment schemes during the first injection series. This is typically the case in registration studies with pre-described fixed injection schemes. We are here, for the first time, presenting real-life frequency data as our patients were studied after at least one year of prior treatment optimisation. We believe that only these data may give patients and injectors an adequate and realistic understanding of the dysphagia risk in BT therapy.

### Reproducibility

Another important novel finding of our study was that in those patients experiencing dysphagia this phenomenon did not occur regularly, but only in about one third of the injection series applied, further reducing the relevance of this phenomenon.

### Severity

Contradictory data in the literature may also be caused by different definitions of dysphagia ranging from dysphagia as an observation (producing high dysphagia frequencies) to dysphagia resulting in functional deficits (with low dysphagia frequencies). For the sake of maximal sensitivity, we used a definition of dysphagia as an observation, thus, including even reports of the mildest forms of dysphagia, well aware that their relevance for the patient's decision to undergo BT therapy may be limited.

### Duration

Whenever dysphagia occurred in our patient series its duration was limited from several days to 2 weeks maximum.

### Predictability

In our study, we evaluated potential risk factors for the occurrence of dysphagia. Due to the proximity of the sternocleidomastoid and scalenii muscles to the pharyngeal muscles, the injection of these muscles is discussed in the literature as a potential risk factor for BT associated dysphagia (Anderson et al. 1992; Comella et al. 1992).That is why we included in our evaluation not only patient age and gender, BT total dose, BT therapy with ABO and BT therapy with anatomical guidance but also BT dose in the sternocleidomastoid muscle, BT dose in the sternocleidomastoid and scalenii muscles, and BT therapy with bilateral sternocleidomastoid muscle injections. None of these factors, however, was related to the dysphagia risk. The trend towards reduced BT total doses, decreased BT doses in the sternocleidomastoid muscle and the sternocleidomastoid and scalenii muscles may have been caused by dose reductions during the dose optimisation phase.

### Ultrasound guidance

Our data indicate that ultrasound guidance does not reduce the overall dysphagia risk, whilst in some patients with dysphagia it may reduce it. Although ultrasound guidance is helpful for BT placement in children and in forearm muscles of adults (especially in writer's cramp where dystonia involvement may be highly selective) (Walter and Dressler 2014), BT placement in CD seems to be equally safe when based on anatomical landmarks and palpation.

## Limitations of the study

The patients’ data were only retrospectively analyzed and the study was not randomized. Furthermore, the occurrence of side effects was documented during the patients’ anamnesis interviews before the next BT injection when these symptoms were no longer present. An objective clinical evaluation of side effects few weeks after the BT injections was not carried out. Besides, the group of patients with dysphagia was markedly smaller than the group of patients without dysphagia as well as the group of ABO treated patients was smaller than the group of INCO or ONA treated patients so that the results have to be interpreted cautiously.

## Conclusions

In CD patients with optimised BT therapy dysphagia may be noticed by one fifth of the patients in about one third of the injection series. It is not associated with a functional deficit and may last several days to 2 weeks. It was not related by patient age or gender, BT total dose, BT dose in the sternocleidomastoid muscle, BT dose in the sternocleidomastoid and scalenii muscles, by BT therapy with bilateral sternocleidomastoid muscle injections, or BT therapy with ABO. Ultrasound guidance is not able to prevent it. Further studies will be necessary to study underlying dystonia associated swallowing abnormalities as a potentially predisposing factor.

## Data Availability

All data are available within the text of the article. Further anonymised data could be made available to qualified investigators upon reasonable request.

## Abbreviations

ABO: AbobotulinumtoxinA
BT: Botulinum toxin
CD: Cervical dystonia
INCO: IncobotulinumtoxinA
MU: Mouse units
SCA: Mm. scalenii
SCM: M. sternocleidomastoideus
ONA: OnabotulinumtoxinA
uMu: Unified mouse units
